# Hybridization of Deep Learning Pre-Trained Models with Machine Learning Classifiers and Fuzzy Min–Max Neural Network for Cervical Cancer Diagnosis

**DOI:** 10.3390/diagnostics13071363

**Published:** 2023-04-06

**Authors:** Madhura Kalbhor, Swati Shinde, Daniela Elena Popescu, D. Jude Hemanth

**Affiliations:** 1 Department of Computer Engineering, Pimpri Chinchwad College of Engineering, Pune 411044, India; 2 Faculty of Electrical Engineering and Information Technology, University of Oradea, 410087 Oradea, Romania; 3 Department of ECE, Karunya Institute of Technology and Sciences, Coimbatore 641114, India

**Keywords:** convolutional neural networks, machine learning, fuzzy min–max neural network (FMMN), cytology image classification, pre-trained models, transfer learning

## Abstract

Medical image analysis and classification is an important application of computer vision wherein disease prediction based on an input image is provided to assist healthcare professionals. There are many deep learning architectures that accept the different medical image modalities and provide the decisions about the diagnosis of various cancers, including breast cancer, cervical cancer, etc. The Pap-smear test is the commonly used diagnostic procedure for early identification of cervical cancer, but it has a high rate of false-positive results due to human error. Therefore, computer-aided diagnostic systems based on deep learning need to be further researched to classify the pap-smear images accurately. A fuzzy min–max neural network is a neuro fuzzy architecture that has many advantages, such as training with a minimum number of passes, handling overlapping class classification, supporting online training and adaptation, etc. This paper has proposed a novel hybrid technique that combines the deep learning architectures with machine learning classifiers and fuzzy min–max neural network for feature extraction and Pap-smear image classification, respectively. The deep learning pretrained models used are Alexnet, ResNet-18, ResNet-50, and GoogleNet. Benchmark datasets used for the experimentation are Herlev and Sipakmed. The highest classification accuracy of 95.33% is obtained using Resnet-50 fine-tuned architecture followed by Alexnet on Sipakmed dataset. In addition to the improved accuracies, the proposed model has utilized the advantages of fuzzy min–max neural network classifiers mentioned in the literature.

## 1. Introduction

Cervical cancer is a type of cancer that develops in the cells of the cervix, which is the lower part of the uterus that connects to the vagina. Cervical cancer is usually caused by a human papillomavirus (HPV) infection, which is a sexually transmitted infection. HPV is a very common virus that can cause abnormal changes in the cells of the cervix, which can eventually lead to cancer if left untreated [1].

**Figure 1:**
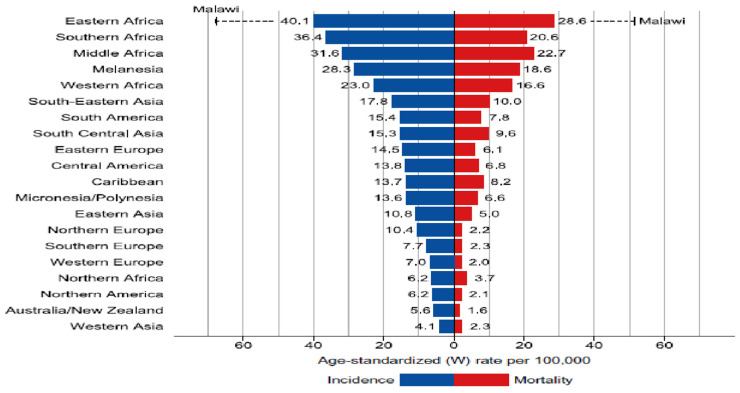
Mortality Age-Standardized Rates and Region-Specific Incidence for Cervical Cancer in 2020. Reprinted with permission from Ref. [1]. Copyright 2020 IARC/WHO.

Cervical carcinoma is the most prevalent cancer diagnosed in 23 countries and the primary cause of mortality in 36 nations [1,2]. Furthermore, 85 percent of cervical cancers were encountered in the late stages. It is the fourth most frequent cancer in women as well as the leading cause of death, with an approximate 604,000 reported incidents and 342,000 deaths worldwide in 2020 [1]. Figure 1 depicts the mortality age-standardized rates and region-specific incidence for cervical cancer in 2020. The (W) world age standardized incidence rate is shown in descending order, and the highest national age-standardized incidence and mortality rates are overlaid. In such areas, it is critical to ensure that resource-intensive vaccination and screening programs are carried out to improve the situation [2].

Pap smear, liquid based cytology, and colposcopy are the main screening methods for cervical cancer diagnosis. In a Pap-smear test, cell samples are collected from the transformation zone of the cervix, and for abnormalities, it is examined under the microscope. The colposcopy examination deals with examining abnormalities in the cervix with the help of the colposcope; it is a direct visual examination done by gynecologists [3]. Regular screening of women over 30 years of age is advisable for early detection and treatment.

The human-based smear analysis is difficult, laborious, time consuming, costly, and prone to errors since each smear slide consists of approximately 3 million cells with varying overlapping and orientation, necessitating the development of a computerized system capable of analyzing the Pap smear effectively and efficiently [4]. Extensive research has been conducted to assist pathologists in tracking cervical cancer with the development of computer-aided diagnostic (CAD) systems. This type of system consists of different steps, including image preprocessing, segmentation, feature extraction, feature selection, and classification. To enhance the image quality, filtering-based preprocessing is carried out. Much work is carried out to segment the nucleus and cytoplasm using different image-processing techniques [5]. The images are used to extract texture, morphological, and color metric features. The feature selection techniques are applied for the identification of the most discriminant features, and then, classifiers are designed to classify the cervical cytology cell images [6]. 

The above mentioned workflow necessitates multiple steps for processing the data. The handcrafted features lack the guarantee superior classification performance, highlighting the inadequacy of automatic learning. Deep learning methods have demonstrated success in a variety of applications over the last decade, including object recognition, natural language processing, signal processing, image classification, segmentation, and so on [7,8,9,10]. The deep network architecture has the ability to learn features automatically based on the spatial relationships among the pixels. The multiple layers with simple nonlinear activation functions are used to transform input data from abstract to specific at multiple levels of feature representation.

The network can learn such hierarchical feature representations from a large scale of training data in an unsupervised or supervised manner. In many practical applications, such learned hierarchical features have outperformed handcrafted designs [11].

Lotfi A. Zadeh [12] proposed a fuzzy logic data analysis approach and an engineering approach. Fuzzy set theory is the basis for fuzzy logic which deals with reasoning that is approximate rather than precise in classical two-valued logic. As a result, it is a technique for formalizing the human capacity for imprecise reasoning. Such reasoning exemplifies the human ability to reason roughly and make decisions in the face of uncertainty [12]. Fuzzy set theory is considered a good framework for classification problems because of the inherent fuzziness in the cluster. FMMN has been used in many applications, including fault detection, lung cancer detection, breast cancer detection, medical data analysis, etc. [13,14,15].

This paper presents a hybrid method for the classification of cytology Pap-smear images into abnormal and normal. The machine learning classifiers and fuzzy min–max neural network are trained for two-class problems using the features to extract by fine tuning the deep learning pre-trained models. The following are the main contributions of the proposed work.

(1) Presents a novel and hybrid approach by leveraging the strengths of pre-trained deep learning models with machine learning classifiers and fuzzy min–max neural networks.

(2) Fine tunes the pretrained CNN architectures, including Alexnet, ResNet-18, ResNet-50, and GoogleNet, to overcome the dataset limitations.

(3) Extracts the learned and specific features from Pap-smear images, which are proven to be more effective than handcrafted features and classify by using different machine learning classifiers and enhancing the classification performance using fuzzy min-max neural network. 

(4) Provides improved accuracy with the advantages of different properties of the fuzzy min–max neural network classifier given by Simpson [16].

## 2. Literature Review

To classify the cervical cytology images, various deep learning and machine learning-based techniques are used, for example, researchers in [17,18] make use of local binary pattern, texture, histogram features, local binary pattern, and grey level features. The features are then given as input to a hybrid classifier system that combines SVM and a neuro-fuzzy for classification of the cervical images [19].

Jyothi Priyankaa et al. (2021) [20] consider Pap smear test images for cancerous cell prediction combined with deep learning techniques for more efficient results. The ResNet50 pre-trained model of convolutional neural networks (CNNs) for the prediction of cancerous cells produces accurate results. Except for the final layer, which is trained according to the requirements, all the layers in the proposed work are considered as they are. This methodology correctly classifies all classes with 74.04 percent accuracy.

Deep transfer learning was used by Anurag Tripathi et al. (2021) [21] to aid in the diagnosis of cervical cancer. They used the SIPAKMED dataset for this purpose. Dyskeratotic, koilocytotic, metaplastic, parabasal, and superficial intermediate were the five classes used. The testing accuracy of ResNet50 is 93.87 percent. The ResNet-152 model achieved an accuracy of 94.89 percent. VGG-16 performed best with parabasal cells, achieving the lowest accuracy of all four models at 92.85 percent. The testing accuracy of VGG-19 was slightly higher than that of VGG-16, which was 94.38 percent.

Wafa Mousser et al. (2019) [22] used deep neural networks and optimized MLP classifiers for the classification of Herlev Pap-smear images. Feature extraction is done using deep neural networks and classification using optimized MLP classifiers. The ability of feature extraction from four different pre-trained models to classify Pap-smear images was investigated. The comparisons concluded that ResNet50 outperforms the VGGs and the InceptionV3 by 15% in Pap-smear image classification.

Kurnianingsih et al. (2019) [23] applied mask R-CNN to the whole slide cell image, outperforming the previous segmentation method in precision, recall, and ZSI. For classification, a VGG-like net is used on whole segmented cells. Results shown for binary classification problem had 98.1% accuracy and for the seven-class problem accuracy of 95.9% is obtained.

Sornapudi et al. (2019) [24] proposed a method for automatically classifying cervical cell images by generating labelled patch data, fine-tuning convolutional neural networks for the extraction of deep hierarchical features and the novel graph-based cell detection approach for cellular level evaluation. The results demonstrated that the proposed pipeline could classify images of single cells as well as overlapping cells. The VGG-19 model performed accurately at classifying cervical cytology patch data, with a precision-recall curve of 95%.

The deep learning approach reviewed in Swati Shinde et al. (2022) [25] can directly process raw images and offers automated learning of features based on specific objective functions, such as detection, segmentation, and classification. Different existing pre-trained models, such as ResNet-50, ResNet-152, and VGG are used in the literature for the classification of Pap-smear images for the diagnosis of cervical cancer. Table 1 shows the summarization of the different papers studied and analyzed.

## 3. Proposed Methodology

**Figure 2:**
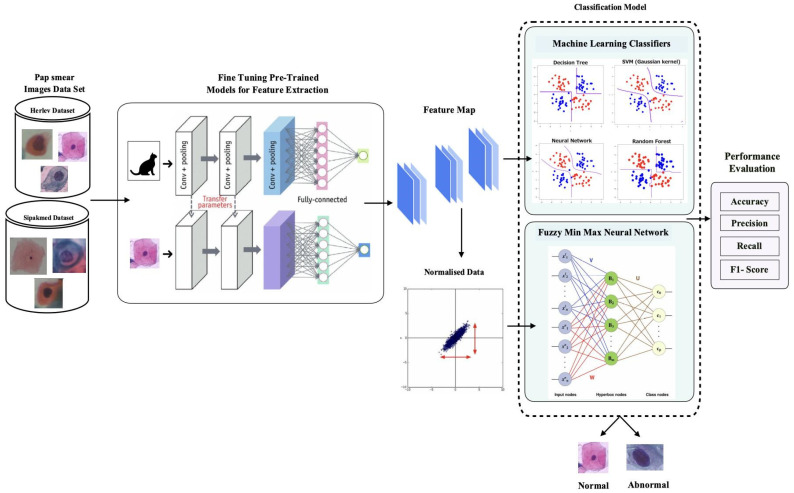
Block diagram of proposed work.

In this paper, a hybrid convolutional neural network classification technique is proposed to classify the cervical cytology images into abnormal and normal. Figure 2 shows the block diagram of the proposed work. The offered hybrid CNN framework is divided into two major phases. In the first phase, a pre-trained deep learning model for feature extraction is used. Successive layers, such as FC6 and FC7, are used to extract features. In the second phase, machine learning classifiers and fuzzy min–max neural network is used for the classification process [27]. 

### 3.1. Module 1 

#### 3.1.1. Feature Extraction Using Pre-Trained Models

For medical image analysis, deep learning architecture is most prevalent. To train a convolutional neural network, a massive quantity of data and high computational resources are required, as well as a longer training time. Transfer learning (TL) is a solution to this problem because it aids in the creation of an accurate model by beginning to learn from previous patterns of knowledge on solving various problems instead of starting from scratch [28,29]. As a result, TL is a technique in artificial intelligence that allows us to transfer knowledge from one model to another [30]. A TL process consists of two steps. 

Step 1: Choose a pre-trained model that is trained on large-scale data that is relevant to the problem at hand.

Step 2: Fine-tune a pre-trained model based on the similarity of our dataset.

AlexNet, GoogleNet, ResNet-18, and ResNet-50 are different pre-trained deep learning architectures that have been experimented with using the proposed hybrid technique. AlexNet, GoogleNet, ResNet-18, and ResNet-50 networks are utilized in the transfer learning process, with the weights pre-trained on the ImageNet dataset [31]. ImageNet is made up of 1 million training images, 50,000 validation images, and 100,000 testing images from 1000 different classes. The earlier layers of the pre-trained models are frozen, which capture more low-level features. Alexnet fc7 layer, ResNet-18 pool 5 layer, ResNet-50 fc1000 layer, and Googlenet loss3-classifier layer are used as features. Figure 2 shows the overall process carried out where feature extraction is carried out using AlexNet. Similarly, GoogleNet, ResNet-18, and ResNet-50 are used. For the machine learning classifiers in Module 2, the number of features is fed for training and testing, as mentioned in Table 2.

#### 3.1.2. Min–Max Normalization

Along with the various machine learning algorithms, the fuzzy min–max neural network is also tested. For classification, the features are normalized and fed into a fuzzy min–max neural network. One of the most common methods for normalizing data is min–max normalization. For each feature, the minimum value is converted to 0, the maximum value is converted to 1, and all other values are converted to a decimal between 0 and 1. The following equation is used to normalize the features [32].

(1) $$X_{new}=\left(X-X_{min}\right)/\left(X_{max}-X_{min}\right)\;$$

where X is the set is of feature values obtained,

$X_{min}$ is minimum value in X, and
$X_{max}$ is maximum value in X.

### 3.2. Module 2

#### 3.2.1. Machine Learning Classifiers

Classification is a machine learning method that determines which class a new object belongs to based on a set of predefined classes. There are numerous classifiers that can be used to classify data, including decision trees, bays, functions, rules, lazy, meta, and so on. In this work we used different classifiers belonging to the different families, and performance comparison is to evaluate the best classifier. We experimented with the BayesNet, Naive Bayes, random forest, random tree, decision table and part machine learning classifiers.

#### 3.2.2. Fuzzy Min–Max Neural Network

Simpson pioneered the hyperboxes for pattern classification [16]. FMM learns using a hyperbox fuzzy set. An expansion parameter theta (θ) controls the size of the hyperbox; in this case the theta (θ) ranges from values 0 to 1. The maximum (max) and minimum (min) points in a hyperbox are used to measure how a training sample accommodates in the hyperbox from a fuzzy membership function [31].

**Figure 3:**
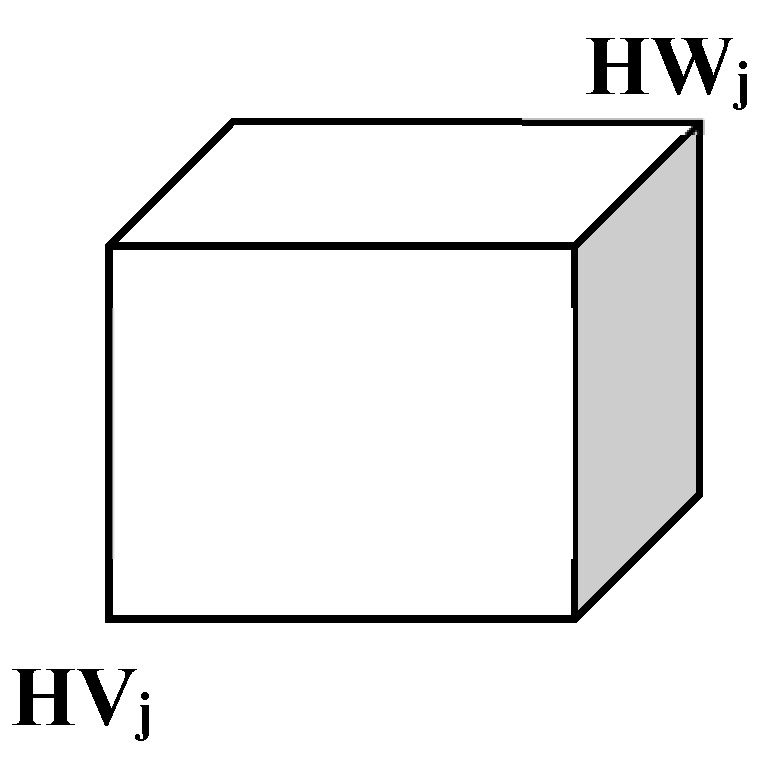
Hyperbox.

Equation (2) defines a hyperbox fuzzy logic with maximum (HW), minimum (HV), and unit hypercube I^n^ points. Figure 3 depicts a 3-D hyperbox with its maximum point (HW_j_) and minimum point (HV_j_).

Fuzzy logic *H_j_* can be used to represent each hyperbox as follows [16]:

(2) $$H_{j}=\left\{HAh,\;HV_{j},\;HW_{j},\;f\left(\left(HAh,HV_{j},\;HW_{j}\right)\right)\right\}\;\;\;\;\;\;\;\forall \;HAh\;\epsilon \;I^{n}$$

where *h*th represents the input pattern as *HA_h_* = (*a_h_*_1_, *a_h_*_2_, …, *a_hn_*). *j*th hyperbox minimum and maximum points are represented as *HV_j_* = (*hv_j_*_1_, *hv_j_*_2_, …, *hv_hn_*) and *HW_j_* = (*hw_j_*_1_, *hw_j_*_2_, …, *hw_hn_*) respectively.

Fuzzy min–max classifier is made up of three layers. The first is input feature vectors (FA), the second is the fuzzy hyperbox sets (FB), and the third is the classification nodes (FC). The fuzzy membership computes the input pattern for various hyperboxes and determines the pattern’s class label. The feature vector obtained from the feature extraction step is provided to the input layer, FA. For hyperboxes, the membership function is evaluated by the nodes (bj) in the fuzzy hyperbox set layer (FB). V and W represent the weights of connections between layers FA and FB, which are a set of min and max points of hyperboxes, respectively. The FMMN expansion process [16] is used to update these parameters. U stores the weights between the nodes in the middle and third layers. Equation (3) shows the U is computed.

(3) $$u_{jk}=\left\{\begin{array}{l} 1\;\;\;\;\;\;\;if\;H_{j}\;is\;hyperbox\;for\;class\;C_{K}\\ 0\;\;\;\;\;\;\;\;\;\;otherwise\;\;\;\;\;\;\;\;\;\;\;\;\;\;\;\;\;\;\;\;\;\;\;\;\;\;\;\;\;\;\;\;\;\;\;\;\; \end{array}\right.$$

FMMN calls the membership function when a new input sample is provided. Equation (4) is used to calculate the membership value.

(4) $$H_{j}{HA}_{h}\;\;\frac{1}{2n}\sum_{i=1}^{n}\left[\;max\left(0,1-max\left(0,\;\gamma \;min\left(1,\;a_{hi}-hw_{ji}\right)\right)\right)+max\left(0,1-max\left(0,\;\gamma \;min\left(1,\;hv_{ji}-a_{hi}\right)\right)\right)\right]{\;}_{\;}$$

where *H_j_* denotes the membership of *j*th hyper box, *HA_h_* is the hth input data, *HW_ji_* is the maximum point of *H_j_*, *HV_ji_* is the minimum point of *Hj*, and γ indicates the sensitivity parameter which controls the decrease in speed of membership value as the gap between *HA_h_* and *H_j_* rises. The FMMN classification method is primarily based on expansion test, overlap test, and contraction test.

##### Expansion

To include a new input pattern, *HA_h_*, in the hyperbox, the following equation is used to determine if a hyperbox can be expanded.

(5) $$n\theta \geq \sum_{i=1}^{n}\left(\max\left(hw_{ji},a_{hi}\right)-\;\min\left(hv_{ji},a_{hi}\right)\right)\;$$

##### Overlap Test

If a hyperbox is chosen for expansion, an overlap test is run to determine whether there is any overlapping between two or more hyperboxes caused by the expansion. If any of the following conditions are met, overlapping of hyperboxes will occur.

Case 1
  (6) $${\mathrm{HV}}_{\mathrm{ji}}<{\mathrm{HV}}_{\mathrm{ki}}<{\mathrm{HW}}_{\mathrm{ji}}<{\mathrm{HW}}_{\mathrm{ki}}\;\;\delta n\;=\;\min({\mathrm{HW}}_{\mathrm{ji}}-{\mathrm{HV}}_{\mathrm{ki}},\;\delta o)$$
Case 2
  (7) $${\mathrm{HV}}_{\mathrm{hi}}<{\mathrm{HV}}_{\mathrm{ki}}<{\mathrm{HW}}_{\mathrm{ki}}<{\mathrm{HW}}_{\mathrm{ji}}\;\;\delta n\;=\;\min({\mathrm{HW}}_{\mathrm{ki}}-{\mathrm{HV}}_{\mathrm{ji}},\;\delta o)$$
Case 3
  (8) $${\mathrm{HV}}_{\mathrm{ji}}<{\mathrm{HV}}_{\mathrm{ki}}<{\mathrm{HW}}_{\mathrm{ki}}<{\mathrm{HW}}_{\mathrm{ji}}\;\;\delta n\;=\;\min(\min({\mathrm{HW}}_{\mathrm{ji}}-{\mathrm{HV}}_{\mathrm{ki}},{\mathrm{HW}}_{\mathrm{ki}}-{\mathrm{HV}}_{\mathrm{ji}}),\;\delta o)$$
Case 4
  (9) $${\mathrm{HV}}_{\mathrm{ki}}<{\mathrm{HV}}_{\mathrm{ji}}<{\mathrm{HW}}_{\mathrm{ji}}<{\mathrm{HW}}_{\mathrm{ki}}\;\;\delta n\;=\;\min(\min({\mathrm{HW}}_{\mathrm{ji}}-{\mathrm{HV}}_{\mathrm{ki}},{\mathrm{HW}}_{\mathrm{ki}}-{\mathrm{HV}}_{\mathrm{ji}}),\;\delta o)$$

##### Contraction

A suitable contraction rule is applied to eliminate the overlap between the hyperboxes if the overlap is detected. The corresponding contraction rules are shown in the following equations with respect to the overlap test rules as stated in the overlap test.

Case 1
  (10) $${\mathrm{HV}}_{j}\Delta <{\mathrm{HV}}_{k}\Delta <{\mathrm{HW}}_{j}\Delta <{\mathrm{HW}}_{k}\Delta <{{\mathrm{HW}}_{j}\Delta }^{\mathrm{new}}={{\mathrm{HW}}_{k}\Delta }^{\mathrm{new}}=({{\mathrm{HW}}_{k}\Delta }^{\mathrm{old}}+{{\mathrm{HW}}_{j}\Delta }^{\mathrm{old}})/2$$
Case 2
  (11) $${\mathrm{HV}}_{k}\Delta <{\mathrm{HV}}_{j}\Delta <{\mathrm{HW}}_{k}\Delta <{\mathrm{HW}}_{j}\Delta <{{\mathrm{HW}}_{k}\Delta }^{\mathrm{new}}={{\mathrm{HV}}_{j}\Delta }^{\mathrm{new}}=({{\mathrm{HW}}_{k}\Delta }^{\mathrm{old}}+{{\mathrm{HW}}_{j}\Delta }^{\mathrm{old}})/2$$
Case 3(a)
  (12) $${\mathrm{HV}}_{j}\Delta <{\mathrm{HV}}_{k}\Delta <{\mathrm{HW}}_{k}\Delta <{\mathrm{HW}}_{j}\Delta \;\mathrm{and}\;({\mathrm{HW}}_{k}\Delta -{\mathrm{HV}}_{j}\Delta )<({\mathrm{HW}}_{j}\Delta -{\mathrm{HV}}_{k}\Delta ),\;{{\mathrm{HV}}_{j}\Delta }^{\mathrm{new}}+{{\mathrm{HW}}_{k}\Delta }^{\mathrm{old}}$$
Case 3(b)
  (13) $${\mathrm{HV}}_{j}\Delta <{\mathrm{HV}}_{k}\Delta <{\mathrm{HW}}_{k}\Delta <{\mathrm{HW}}_{j}\Delta \;\mathrm{and}\;({\mathrm{HW}}_{k}\Delta -{\mathrm{HV}}_{j}\Delta )<({\mathrm{HW}}_{j}\Delta -{\mathrm{HV}}_{k}\Delta ),\;{{\mathrm{HW}}_{j}\Delta }^{\mathrm{new}}+{{\mathrm{HV}}_{k}\Delta }^{\mathrm{old}}$$
Case 4(a)
  (14) $${\mathrm{HV}}_{k}\Delta <{\mathrm{HV}}_{j}\Delta <{\mathrm{HW}}_{j}\Delta <{\mathrm{HW}}_{k}\Delta \;\mathrm{and}\;({\mathrm{HW}}_{k}\Delta -{\mathrm{HV}}_{j}\Delta )<({\mathrm{HW}}_{j}\Delta -{\mathrm{HV}}_{k}\Delta ),\;{{\mathrm{HW}}_{k}\Delta }^{\mathrm{new}}={{\mathrm{HV}}_{j}\Delta }^{\mathrm{old}}$$
Case 4(b)
  (15) $${\mathrm{HV}}_{k}\Delta <{\mathrm{HV}}_{j}\Delta <{\mathrm{HW}}_{j}\Delta <{\mathrm{HW}}_{\mathrm{jk}}\Delta \;\mathrm{and}\;({\mathrm{HW}}_{k}\Delta -{\mathrm{HV}}_{j}\Delta )>({\mathrm{HW}}_{j}\Delta -{\mathrm{HV}}_{k}\Delta ),\;{{\mathrm{HV}}_{j}\Delta }^{\mathrm{new}}={{\mathrm{HW}}_{k}\Delta }^{\mathrm{old}}$$

The training process is completed after successful completion of the preceding three processes, which results in a list of hyperboxes to represent the FMM network.

### 3.3. Algorithm 1

The algorithm for the proposed work is as follows:
**Algorithm 1:** Algorithm for cervical cancer classification  **Input:**  Herlev dataset, Sipakmed dataset of Pap-smear images  **Output:** Prediction of classes—normal or abnormal  **Begin**  **Step 1:** Pre-process the images  **Step 2:** Split the dataset into training and testing datasets  **Step 3:** Pre-trained models= {AlextNet, GoogleNet, ResNet18, ResNet50}  **Step 4:** For each model in Step 3    Train the model    Extract the feature vector  **Step 5:** Classifiers = {{machine learning classifiers: simple logistic, Naive Bays, Bayes Net, decision table, random forest, random tree, PART}, {fuzzy min–max neural network}}  **Step 6:** For each classifier in Step 5    Train with the feature vector    Evaluate with Testing Set  **End**

## 4. Experimentation Environment

The proposed technique is implemented using Matlab software with Intel core i5 processor and 4 GB RAM. To investigate the effectiveness of the proposed techniques, it is applied to two different standard datasets, namely the Herlev dataset and the Sipakmed dataset. Both the datasets are rearranged into two classes, normal and abnormal, and the proposed techniques are used to solve binary classification. The dataset is split into training and testing. 

### 4.1. Herlev Dataset 

**Figure 4:**
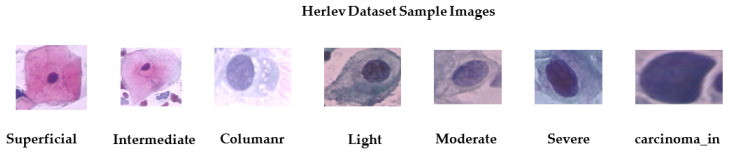
Sample images from Herlev dataset.

It consists of 917 single cell images. Seven classes are converted to normal and abnormal. The normal class contains 242 images, while 675 images belong to the malignant class. Table 3 shows the cell distribution of the dataset and Figure 4 shows sample images from the Herlev dataset [33].

### 4.2. Sipakmed

**Figure 5:**
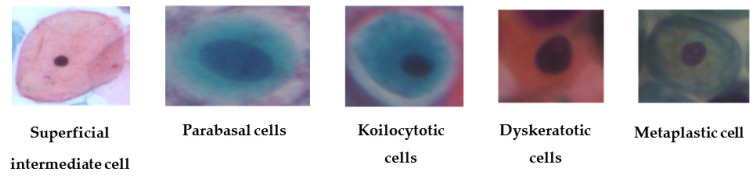
Sample images from Sipakmed dataset.

The Sipakmed dataset consists of 4049 images. There are five categories for classification of the Sipakmed dataset: dyskeratotic, metaplastic, koilocytotic, parabasal, and superficial-intermediate [34]. The Sipakmed dataset samples are shown in Figure 5. Table 4 shows the cell distribution of the dataset.

### 4.3. Performance Measures

Choosing an appropriate evaluation metric is critical for overcoming bias among the various algorithms. Accuracy, sensitivity, specificity, precision and F1 Score are different performance metrics to evaluate the classification performance. True positive (TP) is the number of correctly labelled positive samples, true negative (TN) is the number of correctly classified negative samples, false positive (FP) is the number of negative samples classified as positive, and false negative (FN) is the number of positive instances predicted as negative (FN) [35]. Table 5 shows the formula of evaluation metrics.

## 5. Experiments and Results

The results of an experiment carried out when the AlexNet pretrained model is used as a feature extractor are shown in Table 6. From the results it can be analyzed that the highest classification testing accuracy of 88.6% is given by the simple logistic classifier on the Herlev dataset. With the Sipakmed dataset, 95.14% highest classification accuracy is given by the simple logistic classifier. Hence, the combination of Alexnet with a simple logistic classifier among the experimentations has the best performance.

Experimentation carried out with the GoogleNet pre-trained model results are demonstrated in the following Table 7. Highest testing classification accuracy on Herlev dataset is obtained with simple logistic of 87.32%. On the Sipakmed dataset, the highest accuracy obtained is 92.21% with simple logistic classifiers. With the Googlenet also, the simple logistic is outperforming the other classifiers.

Experimentation carried out with the ResNet-18 pre-trained model results are accumulated in the following Table 8. The highest testing classification accuracies of 88.76% and 93.85% are obtained with the simple logistic classifier on the Herlev and Sipakmed datasets, respectively.

Table 9 shows the experiment carried out when the ResNet-50 pre-trained model is used as a feature extractor. From the results it can be analyzed that the highest classification testing accuracies of 92.03% and 93.60% are given by the simple logistic classifier on the Herlev and Sipakmed datasets, respectively.

Binary classification of cervical cytology images is performed using the pre-trained models, and fuzzy min–max neural networks are elaborated further. Table 10 shows the results of the AlexNet pre-trained model used as a feature extractor. From the tables it can be observed that the highest classification accuracy on the Herlev dataset is 90.22% and good sensitivity of 95% with θ 0.3, whereas the 95.33% is the highest classification accuracy on the Sipakmed dataset and good sensitivity of 95% with θ 0.5. Along with the accuracy, sensitivity, specificity, precision, and F1 score are calculated and presented in the table.

Table 11 represents the results of the Googlenet pre-trained model. From the tables it can be observed that highest classification accuracy on the Herlev dataset is 89.49% and good sensitivity of 97% with θ 0.6, whereas 92.13% is the highest classification accuracy on the Sipakmed dataset and good sensitivity of 91% with θ 0.3.

The results of the RestNet-18 model are shown in Table 12. The highest classification accuracy on the Herlev dataset is 91.67% and good sensitivity of 99% with θ 0.5, whereas 92.87% is the highest classification accuracy on the Sipakmed dataset and good sensitivity of 93% with θ 0.4. 

The results of the RestNet-50 model are shown in Table 13. The highest classification accuracy on the Herlev dataset is 88.77% and good sensitivity of 91%, whereas 95.33% is the highest classification accuracy on the Sipakmed dataset and good sensitivity of 95% with 0 and 0.5, respectively. 

### Performance Analysis

**Figure 6:**
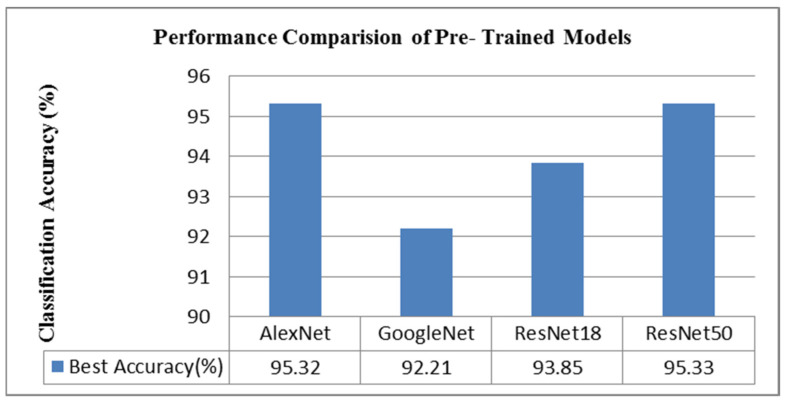
Performance comparison of pre-trained models.

The result analysis discussed above shows that the proposed techniques give overall good classification accuracy. Comparing the performance of the different pretrained models, the best classification accuracy obtained by the experimented pre-trained models is shown in Figure 6. The performance comparison demonstrated with the best classification accuracy, RestNet-50 followed by Alexnet, has performed better than other models with best accuracies of 95.33% and 95.32%, respectively.

The performance comparison between the machine learning classifiers and the FMMN for classification shows that overall, the performance of the FMMN outperforms the machine learning classifier. Table 14 shows the comparative analysis.

**Figure 7:**
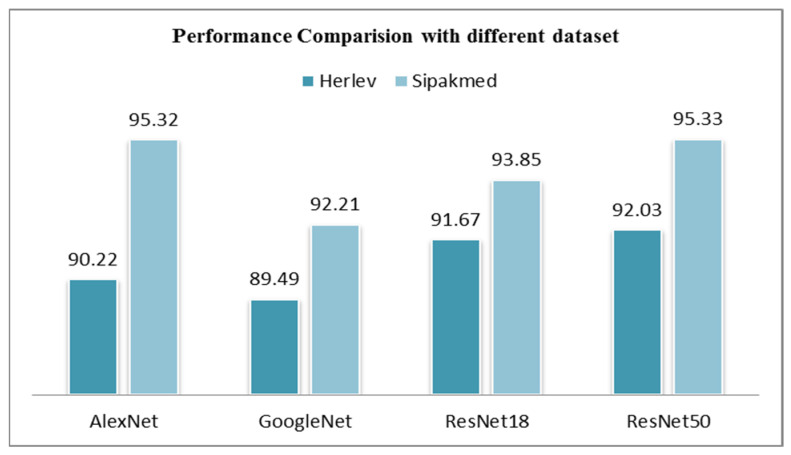
Performance comparison with datasets.

Comparing the two datasets with the classification accuracy obtained, it can be observed from Figure 7 that the Sipakmed dataset average classification accuracy with all the pre-trained models have outperformed over the Herlev dataset. As mentioned, the convolutional neural networks need large amounts of data to train the models, and the Sipakmed dataset has a considerably large number of images as compared to the Herlev dataset. Table 15 shows the comparative study outcomes with the results of the existing studies on cervical cancer diagnosis that uses Pap-smear images using computer-aided applications.

The advantage of the proposed method is it has given a significant good accuracy and sensitivity for the cervical cancer image classification compared with the existing methods. However, the limitation is FMMN is a complex architecture that requires a significant amount of computational resources and training data.

## 6. Conclusions

A novel hybrid deep learning technique is proposed to solve the problem of cervical cytology image classification to aid pathologists to carry out the smear test with good accuracy and less time. The proposed hybrid technique is based on deep learning pretrained models, transfer learning, machine learning classifiers, and fuzzy min–max neural network. Attempts are made to compare the performance of different deep learning models. The highest classification accuracy is given by the ResNet-50 classifier of 95.33% with theta value 0.5. Experimentation is performed on two different datasets to evaluate the performance. Results obtained on the Sipakmed dataset were better than those obtained on the Herlev dataset.

The future scope is to use the modified versions of the fuzzy min–max neural network to improve the classification accuracy. The seven-class, five-class problem for classification can be experimented with the proposed techniques to evaluate the performance for multiclass classification problem.

## Figures and Tables

**Table 1:** Summarization of Prevailing Research Work.

Paper	Data Set	Pre-Processing	Feature Extraction/ Classification	Results
[20]	Herlev University Hospital	Resize, Color to Grey, Expansion of dimensions	RESNET-50	Accuracy 74.04%
[21]	SIPAKMED	Resize 244 × 244	RESNET-50, RESNET-152, VGG-16, VGG-19	Highest 94.89% accuracy was obtained with ResNet-152
[22]	Herlev University Hospital	Data Augmentation	VGG16. InceptionV3 VGG19, ResNet50 Classification—MLP classifier	ResNet-50 89%
[23]	Herlev University Hospital	Data Augmentation Segmentation—Mask R-CNN	VGGNet	Mask R-CNN segmentation produces the best average performance, i.e., 0.92 ± 0.06 precision, 0.91 ± 0.05 recall and 0.91 ± 0.04 ZSI and 0.83 ± 0.10 Binary classification problem 98.1% accuracy Seven-class problem high accuracy of 95.9%
[24]	Herlev University Hospital	Subtraction of blue color space from red color space, skeletonizing and refining boundaries	VGG-19, ResNet-50, DenseNet-120, and Inception_v3	VGG-19—88% Accuracy
[25]	Herlev University Hospital, SIPAKMED, LBC	Data Augmentation	XceptionNet, VGGNet, ResNet50 and Ensemble of classifiers	Accuracy 97%, 99%, and 100%
[26]	Herlev University Hospital	Resize 256 × 256	DCT and Haar transform	Highest 81.11% accuracy was obtained with DCT

**Table 2:** Number of Features Extracted from Pre-Trained Models.

Pre-Trained Model	Alexnet	Googlenet	Resnet-18	Resnet-50
Number of Features	4096	1000	512	1000

**Table 3:** Herlev dataset.

Cell Category		Number of Cells
Normal squamous	Normal	74
Intermediate squamous		70
Columnar		98
Mild dysplasia	Abnormal	182
Moderate dysplasia		146
Severe dysplasia		197
Carcinoma in situ		150
Total		917

**Table 4:** Sipakmed dataset.

Cell Category		Number of Cells
Superficial	Normal	831
Parabasal		787
Koilocytotic	Abnormal	825
Dyskeratotic		813
Metaplastic	Benign	793
Total		4049

**Table 5:** Performance Evaluation Metric.

Assessments	Formula
Accuracy	$\frac{\mathrm{TP}\;+\;\mathrm{TN}}{\mathrm{TP}\;+\;\mathrm{TN}\;+\;\mathrm{FP}\;+\;\mathrm{FN}}$
Sensitivity/Recall	$\frac{\;\mathrm{TP}}{\mathrm{TP}\;+\;\mathrm{FN}}$
Specificity	$\frac{\mathrm{TN}\;}{\mathrm{TN}\;+\;\mathrm{FP}}$
Precision	$\frac{\mathrm{TP}\;}{\mathrm{TP}\;+\;\mathrm{FP}}$
F1 Score	$2\times \frac{\mathrm{Pricision}\;\times \;\mathrm{Recall}\;}{\mathrm{Precision}\;+\;\mathrm{Recall}}$

**Table 6:** Classification accuracy of Alexnet model with machine learning classifiers.

	AlexNet							
Dataset	Classifier	Bayes Net	Navie Bayes	Random Forest	Random Tree	Decision Table	Part	Simple Logistic
Herlev	Testing Accuracy (%)	83.33	82.24	87.68	81.8	88.04	86.59	88.6
Sipakmed		91. 2	91.6	91.2	90.70	93.23	89.5	95.14

**Table 7:** Classification accuracy of Googlenet model with machine learning classifiers.

GoogleNet								
Dataset	Classifier	BayeNet	Navie Bayes	Random Forest	Random Tree	Decision Table	Part	Simple Logistic
Herlev	Testing Accuracy (%)	83.70	82.97	86.96	81.88	84.06	86.59	87.32
Sipakmed		87.37	85.24	90.24	83.11	87.62	89.75	92.21

**Table 8:** Classification accuracy of ResNet-18 model with machine learning classifiers.

ResNet-18								
Dataset	Classifier	BayeNet	Naive Bayes	Random Forest	Random Tree	Decision Table	Part	Simple Logistic
Herlev	Testing Accuracy (%)	86.59	86.59	87.68	82.6	84.42	79.71	88.76
Sipakmed		90.9	89.26	88.36	80.49	84.75	88.42	93.85

**Table 9:** Classification accuracy of ResNet-50 model with machine learning classifiers.

ResNet-50								
Dataset	Classifier	BayeNet	Naive Bayes	Random Forest	Random Tree	Decision Table	Part	Simple Logistic
Herlev	Testing Accuracy (%)	88.04	89.13	88.04	78.62	86.23	81.88	92.03
Sipakmed		89.67	88.19	89.83	81.8	84.75	90	93.60

**Table 10:** Performance Evaluation of Alexnet Pre-Trained Model with Fuzzy Min–Max Neural Network.

		Theta	0	0.1	0.2	0.3	0.4	0.5	0.6	0.7	0.8	0.9	1
Alexnet	Herlev Dataset	Accuracy	87.32	84.06	84.06	90.22	82.97	84.78	85.14	88.04	84.78	39.86	34.78
		Sensitivity	0.90	0.94	0.86	0.95	0.85	0.90	0.91	0.97	0.91	0.19	0.11
		Specificity	0.81	0.58	0.78	0.77	0.77	0.70	0.70	0.64	0.68	0.99	1.00
		Precision	0.93	0.86	0.92	0.92	0.91	0.89	0.89	0.88	0.89	0.97	1.00
		F1 Score	0.91	0.90	0.89	0.93	0.88	0.90	0.90	0.92	0.90	0.31	0.20
	Sipakmed Dataset	Accuracy	92.62	93.20	95.08	95.00	93.93	95.33	94.92	93.69	90.82	80.66	80.00
		Sensitivity	0.95	0.93	0.94	0.94	0.93	0.95	0.95	0.94	0.95	0.99	0.99
		Specificity	0.90	0.93	0.96	0.97	0.95	0.96	0.95	0.93	0.85	0.54	0.52
		Precision	0.93	0.95	0.97	0.98	0.97	0.97	0.97	0.95	0.90	0.76	0.76
		F1 Score	0.94	0.94	0.96	0.96	0.95	0.96	0.96	0.95	0.93	0.86	0.86

**Table 11:** Performance Evaluation of Googlenet Pre-Trained Model with Fuzzy Min–Max Neural Network.

		Theta	0	0.1	0.2	0.3	0.4	0.5	0.6	0.7	0.8	0.9	1
Googlenet	Herlev Dataset	Accuracy	82.25	86.23	83.70	84.78	86.96	88.41	89.49	88.04	86.96	82.25	82.25
		Sensitivity	0.87	0.93	0.89	0.89	0.92	0.98	0.97	0.97	0.95	0.87	0.87
		Specificity	0.68	0.67	0.70	0.74	0.74	0.62	0.70	0.63	0.64	0.70	0.70
		Precision	0.89	0.89	0.89	0.90	0.91	0.88	0.90	0.88	0.88	0.89	0.89
		F1 Score	0.88	0.91	0.89	0.90	0.91	0.93	0.93	0.92	0.91	0.88	0.88
	Sipakmed Dataset	Accuracy	89.34	90.66	90.66	92.13	91.15	91.80	91.15	88.52	85.16	83.03	82.79
		Sensitivity	0.91	0.91	0.92	0.91	0.89	0.91	0.90	0.86	0.86	0.96	0.93
		Specificity	0.86	0.90	0.89	0.94	0.94	0.92	0.93	0.92	0.84	0.64	0.68
		Precision	0.91	0.93	0.93	0.96	0.96	0.95	0.95	0.94	0.89	0.80	0.81
		F1 Score	0.91	0.92	0.92	0.93	0.92	0.93	0.92	0.90	0.87	0.87	0.87

**Table 12:** Performance Evaluation of ResNet-18 Pre-Trained Model with Fuzzy Min–Max Neural Network.

		Theta	0	0.1	0.2	0.3	0.4	0.5	0.6	0.7	0.8	0.9	1
ResNet-18	Herlev	Accuracy	88.77	75.00	89.49	89.13	91.30	91.67	88.04	86.96	86.23	86.96	86.96
		Sensitivity	0.92	0.92	0.91	0.91	0.97	0.99	0.97	0.94	0.94	0.95	0.95
		Specificity	0.81	0.27	0.86	0.85	0.75	0.73	0.64	0.67	0.64	0.66	0.66
		Precision	0.93	0.78	0.95	0.94	0.92	0.91	0.88	0.89	0.88	0.88	0.88
		F1 Score	0.92	0.84	0.93	0.92	0.94	0.95	0.92	0.91	0.91	0.91	0.91
	Sipakmed	Accuracy	91.48	90.82	91.31	92.79	92.87	93.77	90.90	86.80	81.72	77.21	72.46
		Sensitivity	0.93	0.92	0.92	0.92	0.93	0.93	0.93	0.92	0.91	0.93	0.96
		Specificity	0.89	0.88	0.90	0.94	0.93	0.95	0.87	0.79	0.67	0.53	0.36
		Precision	0.93	0.92	0.93	0.96	0.95	0.96	0.92	0.87	0.81	0.75	0.70
		F1 Score	0.93	0.92	0.93	0.94	0.94	0.95	0.93	0.89	0.86	0.83	0.81

**Table 13:** Performance Evaluation of ResNet-50 Pre-Trained Model with Fuzzy Min–Max Neural Network.

		Theta	0	0.1	0.2	0.3	0.4	0.5	0.6	0.7	0.8	0.9	1
ResNet50	Herlev	Accuracy	88.77	86.23	87.32	88.04	87.32	87.32	85.87	87.32	86.96	82.25	81.88
		Sensitivity	0.91	0.93	0.91	0.90	0.90	0.93	0.89	0.93	0.91	0.83	0.85
		Specificity	0.84	0.68	0.78	0.82	0.79	0.73	0.77	0.73	0.77	0.81	0.73
		Precision	0.94	0.89	0.92	0.93	0.92	0.90	0.91	0.90	0.92	0.92	0.90
		F1 Score	0.92	0.91	0.91	0.92	0.91	0.91	0.90	0.91	0.91	0.87	0.87
	Sipakmed	Accuracy	92.05	92.62	92.70	94.18	95.25	95.33	94.18	89.10	84.02	80.82	72.70
		Sensitivity	0.93	0.93	0.94	0.95	0.94	0.95	0.94	0.85	0.82	0.95	0.99
		Specificity	0.90	0.92	0.91	0.93	0.97	0.96	0.95	0.96	0.87	0.60	0.32
		Precision	0.93	0.95	0.94	0.95	0.98	0.97	0.96	0.97	0.91	0.78	0.69
		F1 Score	0.93	0.94	0.94	0.95	0.96	0.96	0.95	0.90	0.86	0.86	0.81

**Table 14:** Best Classification Accuracy (%) of Two Datasets.

	AlexNet	GoogleNet	ResNet18	ResNet50
Herlev	90.22 (FMMN)	89.49 (FMMN)	91.67 (FMMN)	92.03 (Simple logistic)
Sipakmed	95.32 (FMMN)	92.21 (Simple logistic)	93.85 (Simple logistic)	95.33 (FMMN)

**Table 15:** Comparison Between the Proposed Method with the Existing Studies.

Approach	Accuracy
Deep Learning (Resnet-50) [20]	74.04%
ResNet-152 [21]	94.89%
ResNet-50 [22]	89%
VGG-19 [24]	88%
Proposed Model [Hybrid CNN] ResNet50	95.33%

## Data Availability

Data will be available on request to authors.
